# Detection Method for Tomato Leaf Mildew Based on Hyperspectral Fusion Terahertz Technology

**DOI:** 10.3390/foods12030535

**Published:** 2023-01-25

**Authors:** Xiaodong Zhang, Yafei Wang, Zhankun Zhou, Yixue Zhang, Xinzhong Wang

**Affiliations:** 1 College of Agricultural Engineering, Jiangsu University, Zhenjiang 212013, China; 2 Basic Engineering Training Center, Jiangsu University, Zhenjiang 212013, China

**Keywords:** tomato, leaf mildew, terahertz time-domain spectroscopy, near infrared hyperspectral technology, multi-source information fusion

## Abstract

Leaf mildew is a common disease of tomato leaves. Its detection is an important means to reduce yield loss from the disease and improve tomato quality. In this study, a new method was developed for the multi-source detection of tomato leaf mildew by THz hyperspectral imaging through combining internal and external leaf features. First, multi-source information obtained from tomato leaves of different disease grades was extracted by near-infrared hyperspectral imaging and THz time-domain spectroscopy, while the influence of low-frequency noise was removed by the Savitzky Golay (SG) smoothing algorithm. A genetic algorithm (GA) was used to optimize the selection of the characteristic near-infrared hyperspectral band. Principal component analysis (PCA) was employed to optimize the THz characteristic absorption spectra and power spectrum dimensions. Recognition models were developed for different grades of tomato leaf mildew infestation by incorporating near-infrared hyperspectral imaging, THz absorbance, and power spectra using the backpropagation neural network (BPNN), and the models had recognition rates of 95%, 96.67%, and 95%, respectively. Based on the near-infrared hyperspectral features, THz time-domain spectrum features, and classification model, the probability density of the posterior distribution of tomato leaf health parameter variables was recalculated by a Bayesian network model. Finally, a fusion diagnosis and health evaluation model of tomato leaf mildew with hyperspectral fusion THz was established, and the recognition rate of tomato leaf mildew samples reached 97.12%, which improved the recognition accuracy by 0.45% when compared with the single detection method, thereby achieving the accurate detection of facility diseases.

## 1. Introduction

Crop diseases greatly impact the yield and quality of agricultural products, as they can easily cause stem and leaf death, thereby leading to plant decay [1]. In this way, such diseases affect human food security and food safety. Therefore, research on technologies for crop disease diagnosis is of great significance for the early warning and control of these diseases. The traditional diagnosis method used for crop diseases mainly relies on manual diagnosis, which is based on the experience of the examiner. Although this method is simple and convenient, it consumes a great deal of manpower and allows for a high degree of subjectivity, which can lead to misdiagnosis. Currently, the most objective and accurate disease detection methods available are based on laboratory biochemical tests (e.g., the polymerase chain reaction (PCR), nucleic acid hybridization, and DNA microarray techniques) [2,3,4]. Although laboratory-based biochemical detection methods feature the advantage of high identification accuracy, their involved sampling and detection steps require professional operation, are associated with high costs, are lengthy to conduct, and are difficult to conduct on a large-scale [5,6]. In recent years, the rapid development of machine vision and spectral imaging technologies has enabled the quick detection of crop diseases. Such technologies include visible/near-infrared imaging, multispectral/hyperspectral imaging, and chlorophyll fluorescence imaging, which have all been applied to crop disease detection [7,8]. Although this represents progress, most existing studies only discriminate the grade of crop disease by the reflective properties and apparent outer characteristics of the diseased leaves. Because the internal damage of diseased leaves cannot be detected, it remains difficult to achieve the combined analysis of internal and external damage caused by fungal diseases.

In recent years, hyperspectral technology has attracted increasing research interest in the context of disease detection, owing to its merits of featuring high-resolution and integrated mapping. Spectral imaging technology can obtain the spectral image data cubes of the tested sample, thereby accurately obtaining the image information and spectral reflection intensity distribution characteristics of each test sample in each waveband. Fazari et al. [9] established a three-dimensional CNN model using hyperspectral imaging to classify olive anthrax, which performed with a prediction accuracy of 95.73%. Zhang et al. [10] used visible light imaging on downy mildew in combination with machine learning methods to quickly and accurately estimate the severity of cucumber downy mildew in a greenhouse. Image features that had a high correlation with the actual value of greenhouse cucumber downy mildew severity were then used to construct a shallow machine-learning estimation model. The results showed that there was a good linear relationship between the severity of greenhouse cucumber downy mildew estimated by the model and the actual value. Qin et al. [11] proposed a feature band extraction method combining an improved competitive adaptive reweighting algorithm (CARS) and a successive projections algorithm (SPA) with disease information to establish an early detection model of cucumber downy mildew. With this model, the difficult problem of conducting the early detection of cucumber downy mildew was solved.

Terahertz (THz) radiation refers to long wavelength electromagnetic waves with a frequency range of 0.1–10 THz (corresponding to wavelengths of 30 μm–3 mm). THz waves penetrate deeply into the medium and their high correlation helps to determine the exact refractive index and absorption coefficient of a given sample. THz spectroscopy can be utilized to analyze macromolecules and components inside of crops due to the transmission properties of the radiation, which gives it unique advantages in the application of biological information detection. Some researchers have carried out a preliminary attempt at the THz-based detection of crops and agricultural products [12,13]. Di Girolamo et al. [14] imaged 50 chestnuts that were partially infected with Pygmy fungus in the low THz frequency range by means of a homemade 0–0.1 THz small portable imaging system. By assuming different moisture densities and different physical structures of healthy and unhealthy chestnuts, the relationship between the physical parameters (mass or volume) of chestnuts and the light attenuation of healthy and infected chestnuts was tentatively resolved. The results showed that the index of light attenuation combined with the measurement of chestnut weight or volume could successfully identify whether a given chestnut was healthy or diseased. Li et al. [15] employed a recognition model based on a THz spectroscopy technique to analyze data for apple ring rot and cucumber powdery mildew. The researchers established recognition models for common crop diseases based on K-nearest neighbor, SVM, and BP neural network algorithms, respectively, with a correlation coefficient Rp of 0.9649. Their findings demonstrated that hyperspectral and THz technology could be used to detect crop diseases. However, it remains difficult to obtain the internal and external indicators of crop diseases from either external characterization or by using only a single method, and the prediction accuracy also needs to be further improved.

Tomato leaf mold, also known as black mold and black hair, is a tomato disease caused by *Fulvia fulva* (Cooke) Cif. Tomato leaf mildew mainly affects the leaves of infected plants, and in severe cases, also affects the stems, flowers, and fruits. In the early stages of the disease, yellow-green spots with obscure edges appear on the front of affected leaves, while a grayish-white mildew layer appears on the back of the leaves. When the humidity is high, the leaf surface lesions can also grow a mildew layer. After the conidia of tomato leaf mold invade the tomato leaves, they cause changes in the sugars, lipids, proteins, and nucleic acids inside of the leaves. Existing crop disease detection models employ only a single detection method, and such existing methods are unable to fully reflect the condition of the diseased crops. Therefore, this study acquired the near-infrared hyperspectral data, THz power spectrum, and absorbance time-domain spectral data of tomato leaf mildew samples from different infection grades, and carried out a study on a detection model combining both internal and external features of tomato leaf mildew. Through the spectral analysis of tomato leaves under different characteristic frequency bands, a high-precision prediction model of tomato leaf mildew was established.

## 2. Materials and Methods

### 2.1. Experimental Method

**Figure 1:**
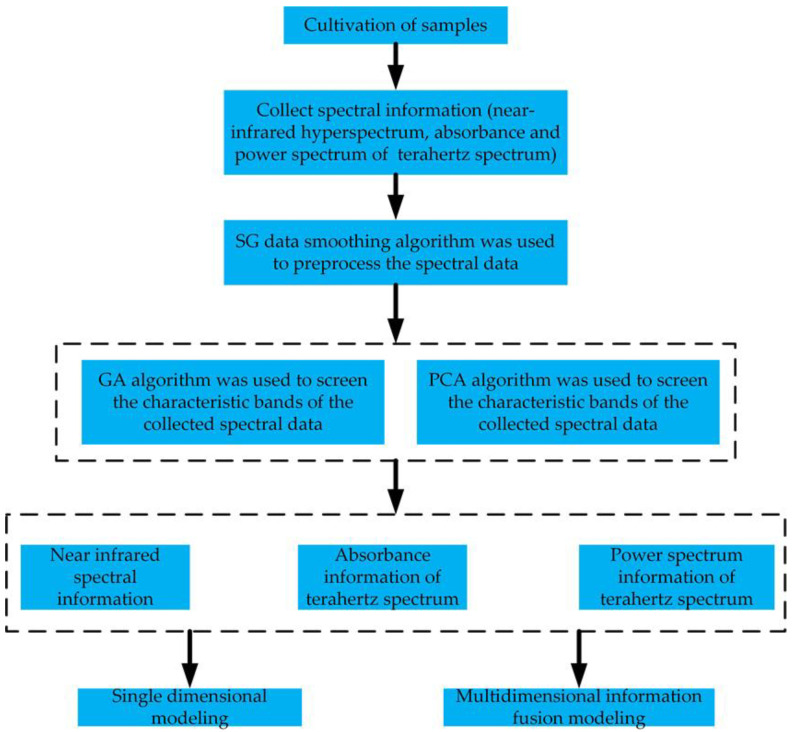
Flow chart of the experiment.

The experiment took tomato leaf mildew samples as the research object and collected test samples with different percentages of diseased spot areas. Using a hyperspectral imaging system and THz time-domain spectral measurement system, the near-infrared spectrum, power spectrum, and absorbance time-domain spectral information of samples with different grades of disease were collected. Algorithm optimization was used to remove interference, remove redundancy, and perform feature extraction. Finally, based on the extracted spectral feature data of different grades of disease, single-dimensional and multi-dimensional fusion tomato leaf mildew recognition models were established, respectively. The specific process is shown in Figure 1.

### 2.2. Cultivation of Samples

Experimental samples of the tomato variety “Cooperative 906” were cultivated in the Venlo greenhouse of Jiangsu University. A 32 × 56 cm rectangular black plastic plate was used for raising the seedlings. Peat, perlite, and vermiculite were mixed to comprise the cultivation substrate, and the seeds were sown in the seedling tray. After budding, the seeds were transplanted into a flowerpot with a diameter of 23.8 cm and a height of 35 cm, and then cultivated by soilless potting using perlite as the substrate nutrient solution.

**Figure 2:**
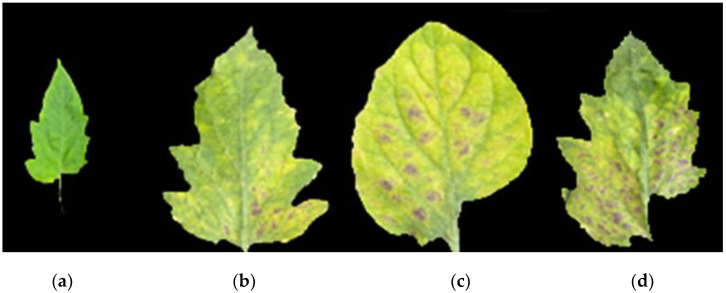
Tomato leaf mildew grades. (**a**) Level 0, (**b**) level 1, (**c**) level 3, (**d**) level 5.

To eliminate interference, a standard concentration of Yamazaki nutrient solution was used to provide the same nutrients for the samples. High temperature and humidity conditions characterized the greenhouse to allow for the development of tomato leaf mildew. After 15 days of infection with tomato leaf mold, the mold was collected from lesioned areas. After collection, the mold was immediately placed into fresh-keeping sealed bags and placed in a portable refrigerated incubator in order to prevent evaporation and minimize the impact of external conditions. Finally, 240 effective samples were obtained, including those obtained from 40 healthy leaves and those obtained from 200 infected leaves. All tomato leaf samples were divided into four disease grades according to GB/T 17980.26-2000. Pictures of these four tomato leaf mildew grades are shown in Figure 2.

Statistics of the effective sample sizes are shown in Table 1. We randomly arranged the samples of the four different tomato leaf mildew grades and randomly divided the training set and prediction set in a proportion of 2:1.

### 2.3. Equipment Used for Experiments

**Figure 3:**
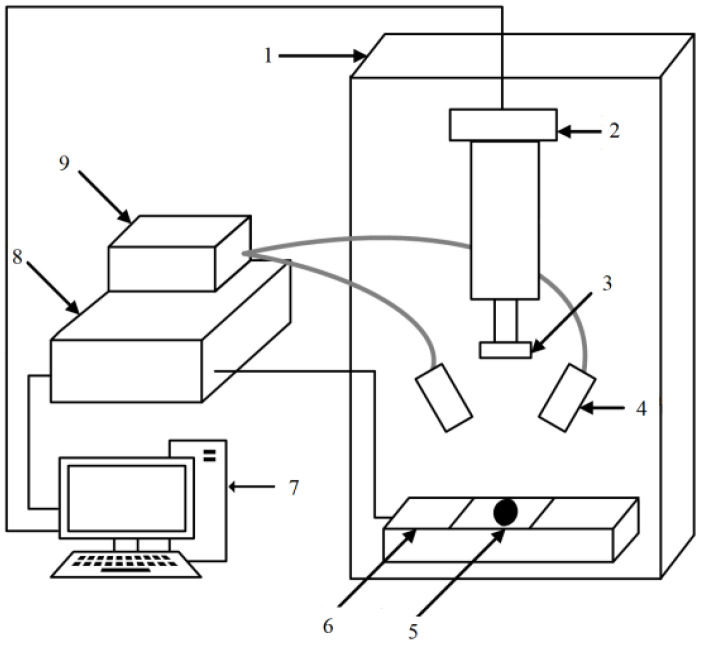
Structure of hyperspectral imaging system. (1) Light box, (2) near-infrared camera, (3) lens, (4) light conduction device, (5) sample, (6) load bearing platform, (7) industrial control machine, (8) displacement control box, (9) light source.

The HIS-VSNIR scanning hyperspectral measurement system (Shanghai Wuling Optoelectronic Technology Co., Ltd.) was used in the experiment. The system is composed of a near-infrared camera (NIR, 871.6–1766.3 nm), ImpectorN17E spectrometer, OLES30 lens, DC adjustable light source, glass fiber symmetrical line light source, stage, self-propelled displacement stage, stepping motor controller, computer, and display. The structure of this hyperspectral imaging system is illustrated in Figure 3.

Conducting pre-sampling tests on tomato leaves is required prior to NIR hyperspectral data acquisition. In order to ensure good clarity and no distortion of the imaging data, the initial exposure scanning time of the hyperspectral imaging system was set to 15 ms, the scanning speed was set to 1.32 mm/s, and the maximum peak reflection imaging intensity of the leaf pre-sampled image data was set to 3000. The dark current generated in the measured sample was required to be calibrated in a black-and-white field in advance, and the reflection intensity range was set to 0–4096. In the sample test, the sample was placed on a full black background separately, and the whole image acquisition and test process was completed in a dark room. The original hyperspectral imaging of the sample was corrected in black and white. The correction formula is as follows:

(1) $$R=\frac{R_{r}-R_{d}}{R_{w}-R_{d}}$$

where *R* is the corrected sample image; *R_r_* is the original image of the sample; *R_d_* is the dark field fixed image; and *R_w_* is the standard whiteboard calibration image.

**Figure 4:**
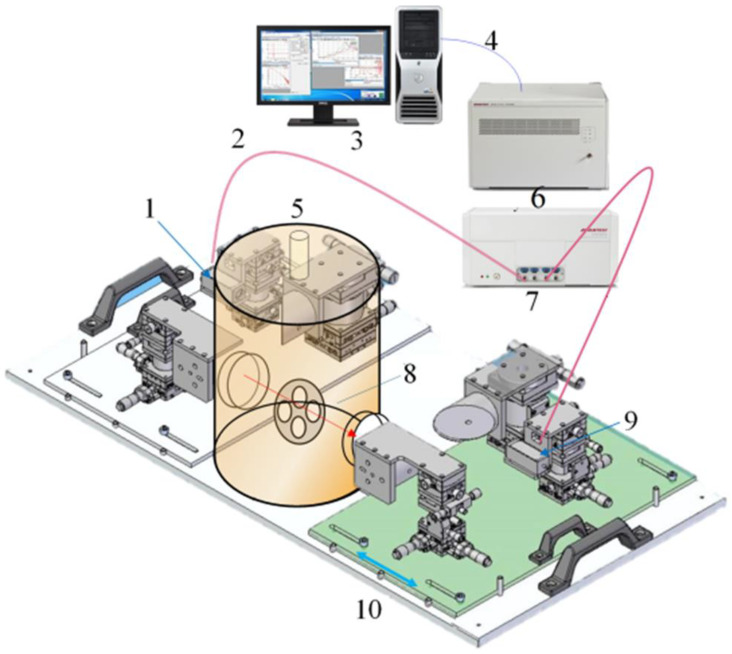
Composition of the measuring system. (1) THz transmitter, (2) optical fiber, (3) operation/analysis computer, (4) ethernet, (5) low-temperature thermostat transmission module, (6) analysis unit, (7) measuring unit, (8) sample stage, (9) THz detector, (10) movable support.

In this experiment, the TS7400 THz time-domain spectral measurement system (Advantest Corporation of Japan) was used to collect the THz information of samples, which was specially customized for the detection of agricultural biological information. A structure introduction diagram of the THz time-domain spectrum measurement system is shown in Figure 4.

The measurement range of the TS7400 THz time-domain spectral measurement system was 0.1–4.0 THz and the frequency sampling interval selected for testing was 0.0038 THz, which can be used to detect 225 cm^2^ samples. This meets the detection requirements of tomato leaves. In order to improve the accuracy of the acquired data and reduce the effect of moisture on the THz time-domain spectrum, before scanning the tomato samples, the tomato leaves were first freeze-dried using a vacuum freeze-dryer set to −65°C and then left for 36 h to reduce their moisture content to less than 3%. Additionally, the THz time-domain spectral scanning cabinet was filled with nitrogen to keep the maximum relative humidity below 5%.

In order to obtain the best response information for tomato leaf mildew samples, this study used the power spectrum and absorbance information for sample analysis. ‘Power spectrum’ is an abbreviation for the power spectrum density function, which is defined as the signal power within the unit frequency band. It represents the variation of the signal power with frequency, i.e., the distribution of the signal power in the frequency domain. Absorbance is used to express the degree of light absorption by substances. Samples of different grades of tomato leaf mildew have different absorbances.

### 2.4. Data Processing

#### 2.4.1. Data Smoothing

**Figure 5:**
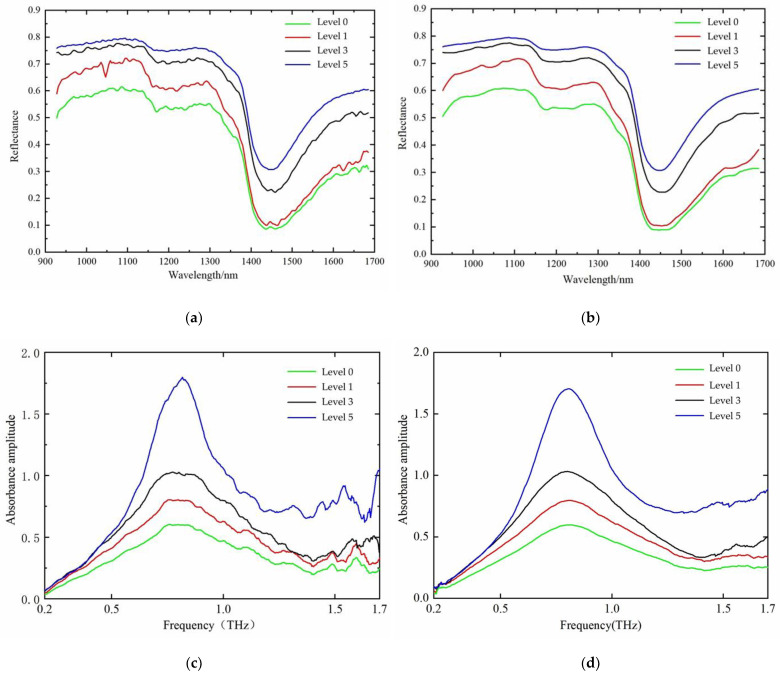

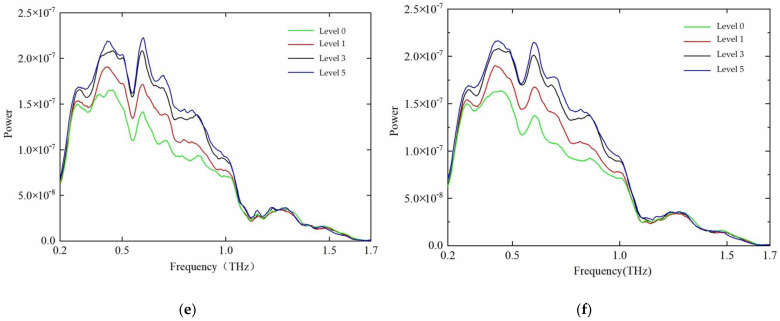
Data of tomato leaf mildew samples before and after SG smoothing preprocessing. (**a**) Near-infrared primary spectrum, (**b**) near-infrared spectra after SG smoothing, (**c**) THz absorbance spectrum, (**d**) THz absorbance spectrum after SG smoothing, (**e**) THz power spectrum, (**f**) THz power spectrum after SG smoothing.

The SG smoothing algorithm is commonly used in data pre-processing, which features the advantages of being simple, convenient, fast, and efficient [16]. The principle of the algorithm is to first take a window with an odd number of points in width, use the least squares method to fit through the translation of the window, and then replace the original value with the fitting value of the point in the window to achieve the effect of smoothing the data. In this study, the SG smoothing algorithm was used to preprocess the data, and the window width was 7 points/time. This algorithm can be used to effectively reduce interference signals and improve both modeling efficiency and accuracy. After the above preprocessing, the before-and-after data comparison of the spectral data of tomato leaf mildew samples was obtained, as shown in Figure 5.

#### 2.4.2. Characteristic Band screening

Because the collected spectral data contains many redundant and collinear information characteristics, this interferes with the extraction of effective spectral information, consequently leading to the effective spectral information extraction model being too complex and hence difficult to calculate. In this paper, a genetic algorithm (GA) and principal component analysis (PCA) were used to select the characteristic wavelength in order to reduce the influence of information redundancy and collinearity, simplify the model, and reduce the amount of calculation. The use of a GA algorithm represents an intelligent optimization method that simulates the evolutionary process that occurs by the natural selection of organisms [17]. When running the GA to screen the near-infrared hyperspectral characteristic bands in the current study, the crossover probability was set to 0.5, the population size was set to 30, and the mutation probability was 0.01. The characteristic wavelength was determined as the wavelength with the highest frequency of 100 GA iterations.

PCA is a multivariate statistical method used for analyzing correlations among multiple variables. The method converts a group of variables that may correlate with a group of linearly unrelated variables through orthogonal transformation [18]. The new variables obtained through PCA can reduce the number of variables while preserving the original feature information as much as possible. Therefore, PCA is a suitable method for the dimension reduction and feature extraction of THz time-domain spectral data.

#### 2.4.3. Establishment of the Model

The backpropagation neural network (BPNN) is a powerful learning system that can realize highly nonlinear mapping between the input and output [19]. The number of units in the input layer of the BPNN model is the number of principal component feature variables, while its output layer is the disease spot area percentage; that is, the grade of tomato leaf mildew in this study. The non-linear Sigmoid type function was selected as the action function of the model, the learning rate was set to 0.6, the number of iterations was set to 300, the target deviation was set to 10–5, and other settings were kept as the default settings of the MATLAB self-contained toolbox. The activation function of the hidden layer was tansig and the activation function of the output layer was purelin.

Bayesian reasoning is a commonly used method of statistical reasoning. The main way to obtain information and evidence is by the updating of probability assumptions by the Bayesian theorem [20]. The steps for the classification and recognition of tomato leaf mildew samples by Bayesian reasoning are as follows.

(1) Calculate the prior probability; that is, the proportion of each level in the tomato leaf mildew sample. The prior probability formula is as shown below:


(2) $$P\left(Y=c_{k}\right)=\frac{\sum_{i=1}^{N}\left(y_{i}=c_{k}\right)}{N},k=1,2,\cdots ,K$$

(2) Calculate the conditional probability; that is, the conditional probability of each attribute in the training data set:


(3) $$P\left(X^{\left(j\right)}=a_{jl}|Y=C_{k}\right)=\frac{\sum_{i=1}^{N}I\left(X_{i}^{\left(j\right)}=a_{jl},y_{i}=c_{k}\right)}{\sum_{i=1}^{N}I\left(y_{i}=c_{k}\right)}\;j=1,2,\cdots ,n,l=1,2,\cdots ,s_{j^{\prime }}k=1,2,\cdots ,K$$

(3) For a given example $x_{i}={\left(x^{\left(1\right)},x^{\left(2\right)},\cdots ,x^{\left(n\right)}\right)}^{T}$, *a posteriori* probability is calculated.

(4) Calculate the maximum *a posteriori* probability and determine the class of instance *x* according to the value of the maximum *a posteriori* probability:


(4) $$y=\arg\underset{c_{k}}{max}P\left(Y=c_{k}\right)\overset{n}{\underset{j=1}{\prod }}P\left(X^{\left(j\right)}=x^{\left(j\right)}|Y=c_{k}\right)$$

There are three types of node variables in the Bayesian network model: hyperspectral characteristic band nodes representing the health status of tomato leaves $f_{a}=\left\{f_{a1},f_{a2},\cdots ,f_{\mathrm{aN}}\right\}$, THz characteristic band nodes representing the health status of tomato leaves $f_{b}=\left\{f_{b1},f_{b2},\cdots ,f_{\mathrm{bN}}\right\}$, and parameter nodes representing the health status of tomato leaves $Y=\left\{Y_{1},Y_{2},\cdots ,Y_{M}\right\}$. The functional relationship between hyperspectral, TH, and parameter characteristic band nodes representing the health status of tomato leaves is as shown below:


(5) $$Y=F\left(u,f_{a},f_{b}\right)$$

**Figure 6:**
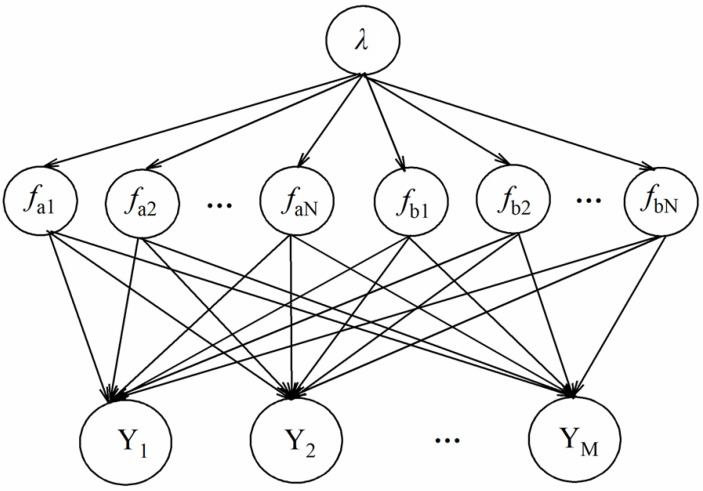
Improved Bayesian network model for the health state analysis of tomato leaves.

After introducing the new node λ, the health status analysis of tomato leaves based on the Bayesian network model is obtained, as shown in Figure 6. Bayesian networks can be introduced by virtue of the prior distribution of health parameters. In the Bayesian network model, λ is the percentage of the diseased spot area; that is, the threshold value, which is set to 0.5.

## 3. Results and Discussion

### 3.1. Screening of Near-Infrared Hyperspectral Characteristic Bands

**Figure 7:**
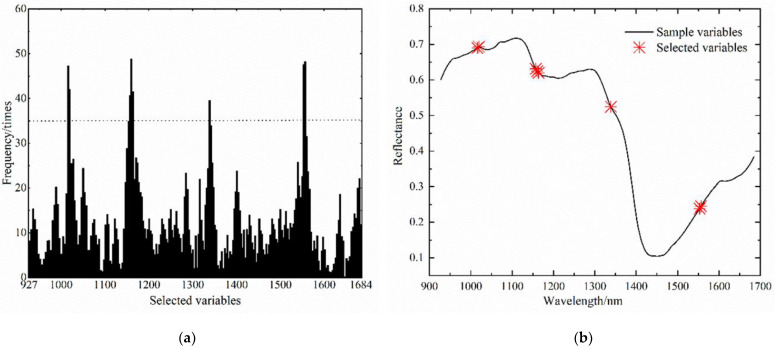
Running process of the genetic algorithm. (**a**) Selected times of each wavelength point during genetic iteration, (**b**) schematic diagram of characteristic bands screened by the genetic algorithm.

Figure 7 shows the selected frequency of each variable of the tomato leaf mildew samples. The variables that were selected more than 35 times became the final selected variables, and GA greatly reduced the number of variables from hundreds to only several. The GA operation screened eight near-infrared hyper-spectral characteristic wavebands of tomato leaf mildew samples, which corresponded to 1016 nm, 1019.9 nm, 1157.1 nm, 1160.5 nm, 1163.9 nm, 1338.7 nm, 1553.3 nm, and 1556.7 nm, respectively.

### 3.2. Terahertz Time-Domain Spectral Data Processing Results

#### 3.2.1. Terahertz Time-Domain Spectral Analysis

The average values of the sample power spectrum and absorbance spectrum can be obtained by THz time-domain spectroscopy. Figure 5e shows the average value curve of the power spectrum of the four tomato leaf mildew grades at 0.1–2.0 THz, with clear absorption peaks observed at approximately 0.43 THz and 1.27 THz, as well as a faint absorption peak at approximately 0.53 THz. Figure 5c shows the mean absorbance curves for the four tomato leaf mildew classes at 0.1–2.0 THz, with a clear absorption peak observed at approximately 0.79 THz. For level 3 mold leaves, a relatively clear absorption peak was observed at approximately 1.89 THz. However, the other three grades of leaf mildew in leaves did not have this absorption peak, indicating that this absorption may be an error caused by the equipment itself, and hence should not be directly judged as the peak of the absorbance sample. The identification of each sample should be achieved by mathematical modeling.

**Figure 8:**
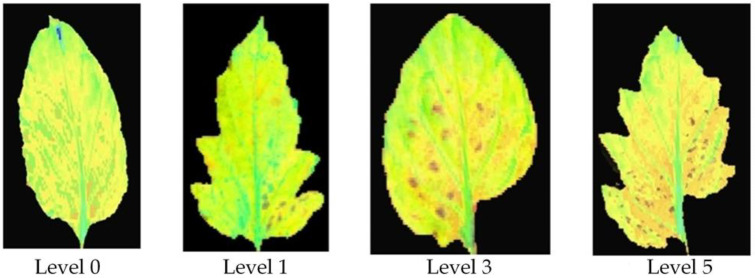
Terahertz images of tomato leaves with different disease grades.

Figure 8 shows the THz frequency domain image at 0.4 THz derived from the data distribution. It can be seen that the difference between the diseased and healthy areas of the leaves is reflected by the color information corresponding to the strength of the frequency domain values, which indicates that the processed THz feature image can reflect the changes in crops from a visual perspective.

#### 3.2.2. Screening of the Terahertz Time-Domain Spectrum Characteristic Frequency Band

**Figure 9:**
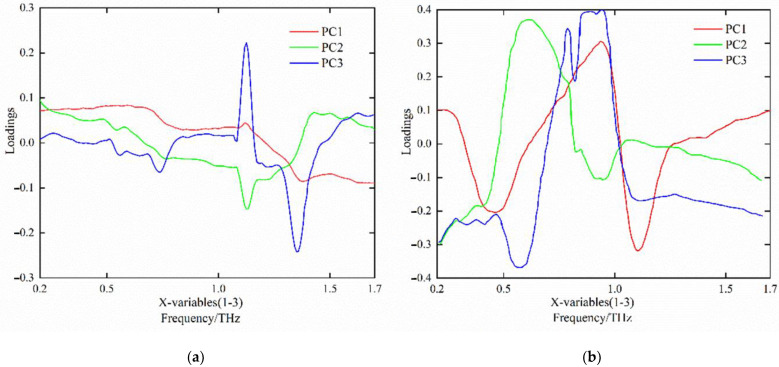
Load curves of the first three principal components of tomato leaf mildew samples. (**a**) absorbance dimension, (**b**) power dimension.

PCA enables the original spectral bands to obtain principal components through linear combination, and also determines the characteristic wavelength according to the absolute value of the loadings of the principal components. The loading refers to the correlation coefficient between the principal component and the original wavelength variable, which is used to reflect the closeness degree between the principal component and each wavelength variable [21]. Loading curves of the first three principal components of tomato leaf mildew samples are shown below in Figure 9. The absolute value of loadings at the peak and trough of each principal component curve was large and the corresponding wavelength was the characteristic wavelength. Therefore, after smoothing the power spectrum, five characteristic wavelengths were obtained: 0.413 THz, 0.752 THz, 1.394 THz, 1.457 THz, and 1.622 THz, respectively. Using the same method, the smoothed absorbance spectrum obtained six characteristic wavelengths: 0.249 THz, 0.567 THz, 0.813 THz, 1.243 THz, 1.771 THz, and 1.892 THz, respectively. 

**Figure 10:**
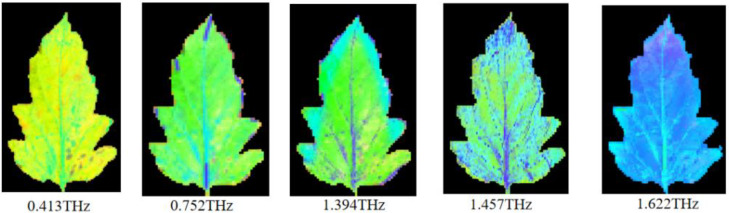
Terahertz time-domain spectral characteristic image.

To further compare the visualized images in different frequency domains, THz frequency domain imaging was performed for five characteristic spectra, as shown in Figure 10. The images of the samples were relatively distinct at the 0.413 THz, 0.752 THz, and 1.394 THz frequencies. At the frequency of 0.413 THz, the image of the sample was the clearest and the recognition effect was the best. However, at the 1.457 THz and 1.622 THz frequencies, the sample images became blurred.

The PCA method was used to establish the identification model of different tomato leaf mildew grades on the power spectrum dimension and the absorbance dimension of the THz time-domain spectrum. Table 2 shows the PCA results of the spectral data in both dimensions combined with the preprocessing of the SG smoothing algorithm. As shown in Table 2, the cumulative variance contribution of the first two principal components (PC1 and PC2) to the level variable of tomato leaf mildew was above 85% [22]. Hence, PC1 and PC2 were selected for the analysis.

**Figure 11:**
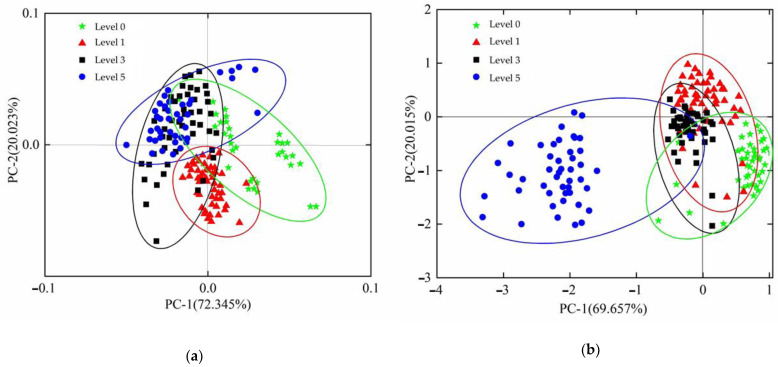
Scatter diagram of tomato leaf mildew sample distribution. (**a**) absorbance scatter, (**b**) power scatter.

According to Figure 11, it can be seen that the confidence ellipse of the absorbance data of different grades of tomato leaf mildew exhibited an intertwined state with a discrimination rate of 19.8%. This is because the recognition rate of level 1 grade tomato leaves was 84.9%, while the recognition rates of tomato leaves classed as grades 0, 3, and 5 were lower. The confidence ellipse of the power spectrum data of different grades of tomato leaf mildew also exhibited an intertwined state, with a discrimination rate of 24.7%. The above results show that the recognition rate of tomato leaf mildew using the SG smoothing preprocessing algorithm combined with the PCA model was low, and that the PCA method could not fully mine the spectral information of tomato leaves with different disease grades. Hence, it is necessary that other algorithms are used to build models to improve the prediction accuracy.

### 3.3. Single-Model Analysis

After using the GA and PCA algorithms to reduce the dimension of the data and screen the characteristic variables, a prediction model of tomato leaf mildew disease was developed based on the screened feature variables by the BPNN method. Before the model was established, it was necessary to carry out PCA and extract the sub-vectors of the principal components to form the input of pattern recognition. During the training process of the model, the number of principal component variables affects both the accuracy and stability of the model. Too few principal component factors will lead to excessive loss of information and reduce the accuracy of the model. However, if the number of principal component factors is too great, an excessive amount of redundant information will be introduced, which both influences the robustness of the model and lengthens the data processing time [22]. Therefore, it is important to select the appropriate number of principal component factors for the establishment of the model.

**Figure 12:**
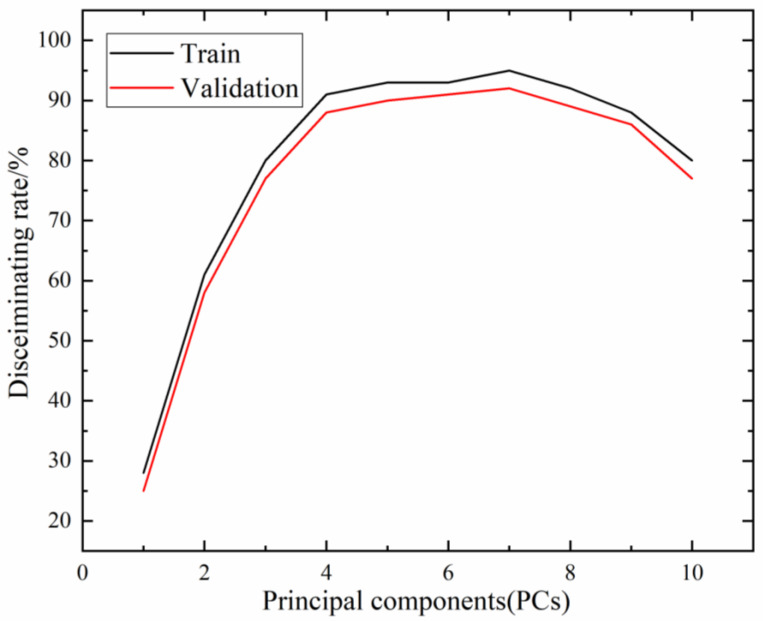
Recognition results of training and prediction under different principal component factors.

Figure 12 shows the recognition results of the BPNN model training and prediction under different numbers of principal component factors. It can be seen that, initially, with the increasing number of principal component factors, the recognition rates in the training and prediction sets generally exhibited an increasing trend, while after the number of principal component factors reached 7, the recognition rates of the models stabilized, and then even exhibited a moderately decreasing trend.

**Figure 13:**
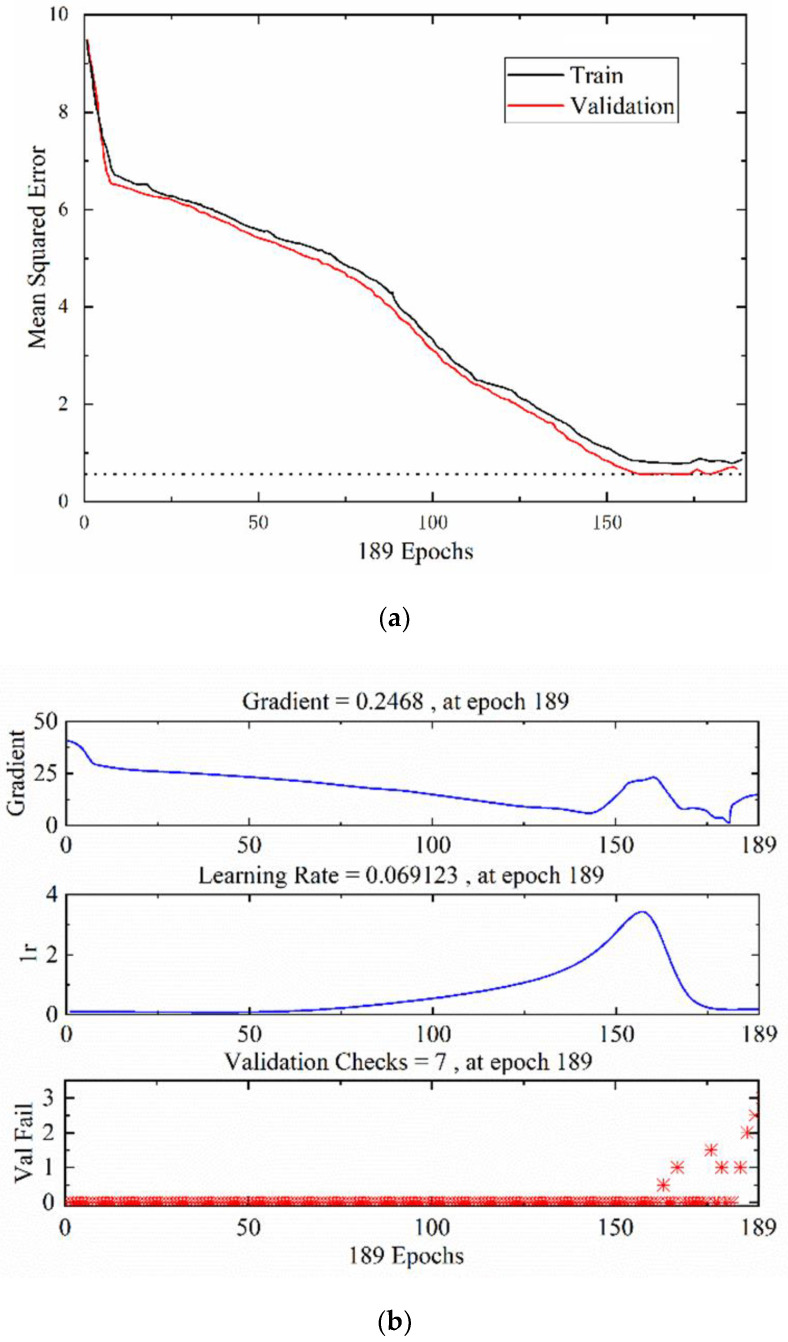

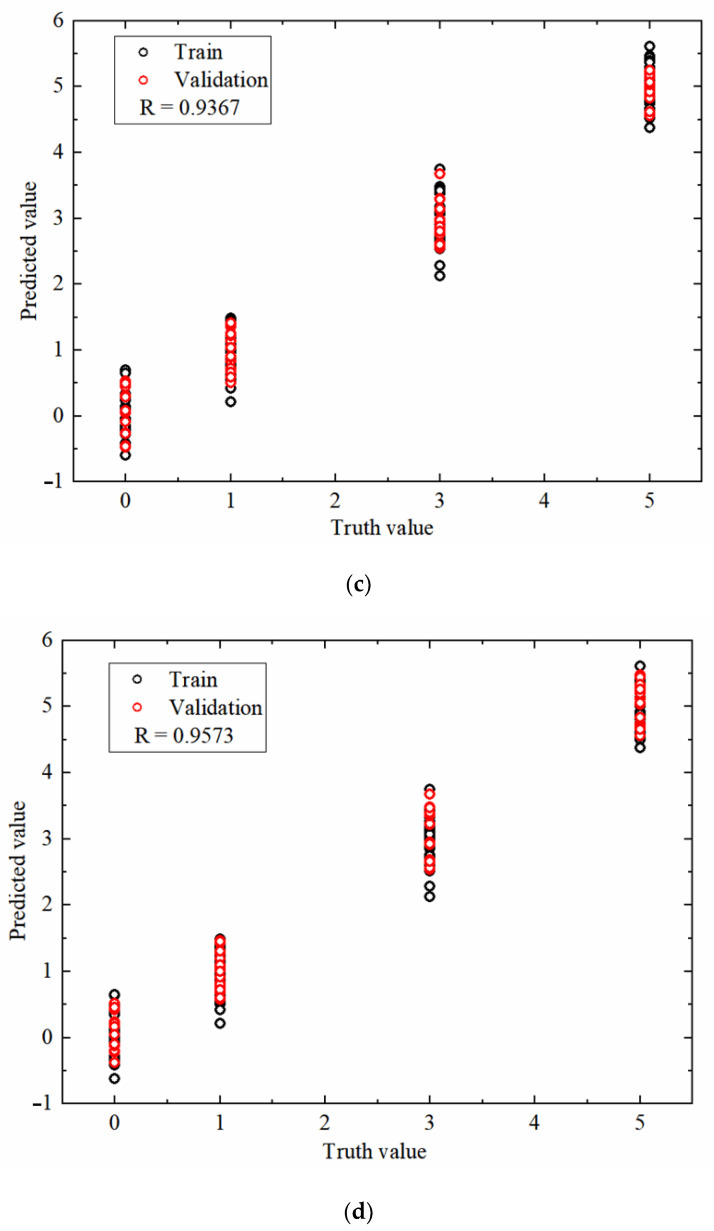

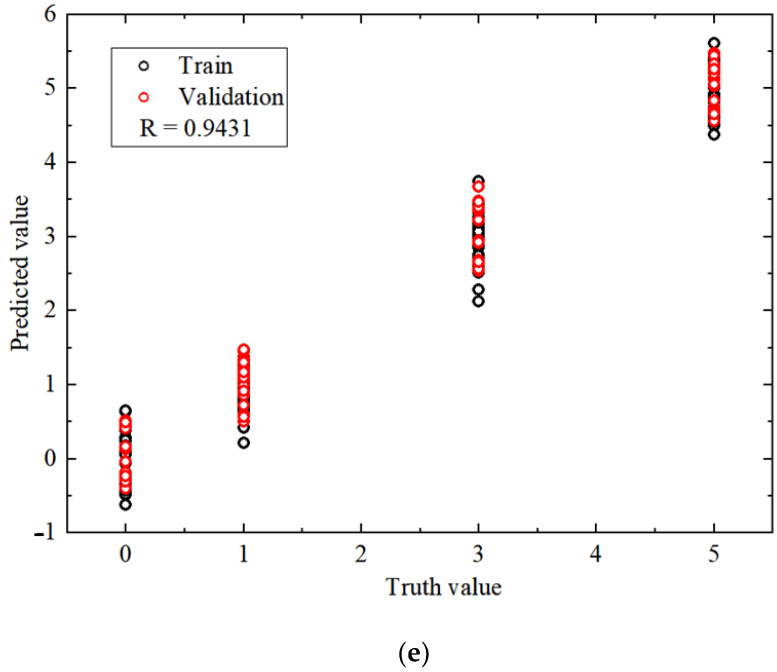
(**a**) Performance diagram of the backpropagation neural network, (**b**) training status of the backpropagation neural network, (**c**) regression analysis of the backpropagation neural network using the near-infrared hyperspectrum, (**d**) regression analysis of the backpropagation neural network using the THz absorbance, (**e**) regression analysis of the backpropagation neural network using the THz power spectrum.

Figure 13a shows the BPNN performance graph, which shows that the minimum MSE was 0.6792. Figure 13b shows the BPNN training status graph, which shows that the actual training times were 189. Figure 13c–e shows the BPNN regression analysis graph. When the test set classification index falls within the threshold of the training set classification index, the recognition result is correct. The converse indicates that the classification recognition is incorrect. The precision of the proposed model under the near-infrared hyperspectrum was determined to be R = 0.9367, while under the THz absorbance dimension it was R = 0.9573, and under the THz power spectrum dimension it was R = 0.9431. Based on the actual classification diagram and prediction classification diagram of all the test sets, it was found that the BPNN model was able to identify almost all tomato leaves with leaf mildew.

To evaluate the detection accuracy of the model, this study comprehensively evaluated the recognition results with the recognition accuracy variable *P*, which is an indicator used to measure the detection signal-to-noise ratio; that is, the percentage of the ‘correct’ detection results among all detection results. The calculation formula is shown below [23]:

(6) $$P=\frac{T_{P}}{T_{P}+F_{P}}$$

where *T_P_* represents the correctly identified tomato leaf mildew samples, and *F_P_* represents the incorrectly identified tomato leaf mildew samples.

In this study, tomato leaf mildew was divided into four grades, so the prediction accuracy of each level was taken as the evaluation index used for statistics. The results are shown in Table 3.

The results show that in the model established by the characteristic variables, the overall detection accuracy of the samples was more than 90%, featuring high accuracy. The highest and lowest detection accuracy rates for the Level 1 samples were 96% and 92%, respectively. The average accuracy rate was 94.67%. Compared to Level 3, the recognition effect in Level 1 was better. Compared to Level 5, the recognition rate was slightly lower. Each model had the highest detection accuracy rate for the Level 0 samples. Hence, the PCA-BPNN model of the power spectrum dimension is the optimal model for comprehensive evaluation. Its prediction accuracy for grades 0, 1, 2, 3, and 4 was 100%, 96%, 95.45%, and 94.74%, respectively, with an overall prediction accuracy of 96.67%.

### 3.4. Fusion Model Analysis

**Figure 14:**
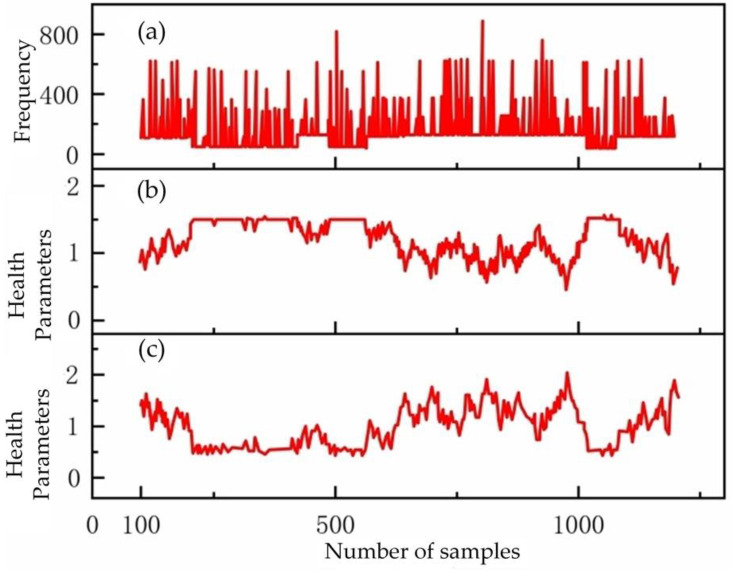
Gibbs sampling diagram. (**a**) represents the frequency of tomato leaves infected with leaf mildew, while (**b**,**c**) each represent a health parameter map of a hyperspectral THz characteristic band.

Figure 14 shows the Gibbs sampling dynamics of the health parameters under the condition of tomato leaves infected with leaf mildew. Figure 14a represents the frequency of tomato leaves infected with leaf mildew, while Figure 14b,c each represent a health parameter map of a hyperspectral THz characteristic band. 

**Figure 15:**
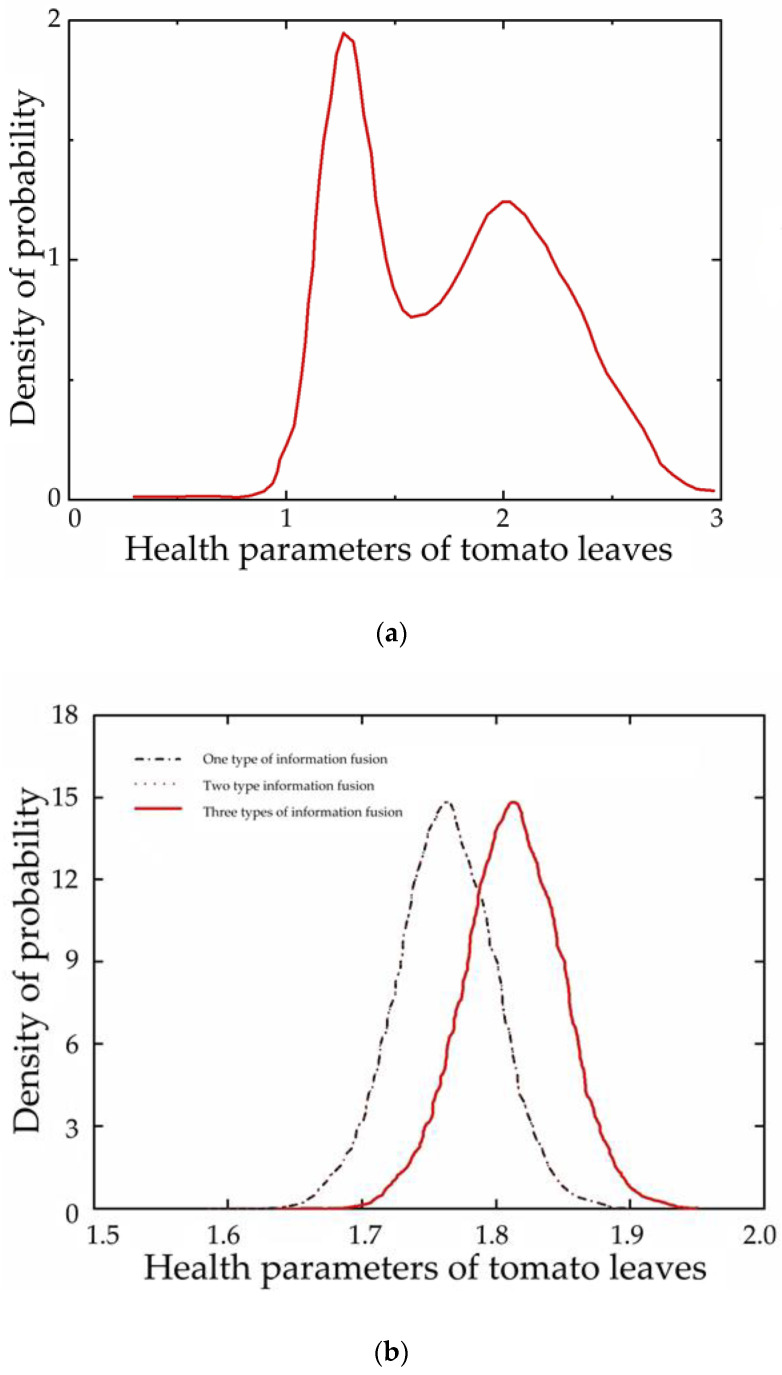
Schematic diagram of the probability density of health parameters. (**a**) Posterior distribution of changes in health parameters, (**b**) posterior distribution of changes in health parameters after information fusion.

In Figure 15, a probability density diagram was used to characterize the leaf health parameters of tomato leaf mold. Type I information fusion refers to THz spectral absorbance feature band fusion, while type II information fusion refers to THz spectral power spectrum feature band fusion, and type III information fusion refers to hyperspectral feature band fusion. These three types of information are fused to re-evaluate the health parameter indicators and calculate the recognition rate. After fusing the three types of prior information, it can be seen from the figure that the estimation results were significantly improved after fusing type I information. The posterior distribution of tomato pests and diseased leaves illustrates this point more clearly. The health parameters of tomato leaf mildew posterior samples were also all distributed around 1.75, indicating that the modified Bayesian network model is effective in identifying tomato leaf mildew samples. After the fusion of the prior information, the variables and the actual values increased in agreement, and the final obtained health parameters and posterior distribution of tomato leaves in the state of infection with pests and disease were very close to the actual values. 

As shown in Table 4, the overall recognition rate of the improved Bayesian inference for tomato leaf mildew was finally obtained as 97.12%. Therefore, the hyperspectral fusion THz-based technique is feasible for application in tomato leaf mildew recognition.

## 4. Conclusions

In this study, a new method was proposed for the multi-source detection of tomato leaf mildew by THz hyperspectral imaging through the fusion of internal and external features. First, multi-source information obtained from diseased tomato leaves of different grades was extracted by near-infrared hyperspectral imaging and THz time-domain spectroscopy, while the influence of low-frequency noise was removed by the Savitzky Golay (SG) smoothing algorithm. A genetic algorithm (GA) was used to optimize the characteristic near-infrared hyperspectral band. Principal component analysis (PCA) was employed to optimize the THz characteristic absorption spectra and power spectrum dimensions. Based on the near-infrared hyperspectral features, THz time-domain spectrum features, and classification model, the probability density of the posterior distribution of tomato leaf health parameter variables was recalculated by the use of the Bayesian network. Finally, a fusion diagnosis and health evaluation model of tomato leaf mildew using hyperspectral THz was established, and the recognition rate of tomato leaf mildew samples reached 97.12%. This study has therefore successfully developed a method to realize the detection of tomato leaf mildew which can provide a scientific basis for the subsequent monitoring of the disease and provide theoretical support for the development of disease detection instruments.

## Figures and Tables

**Table 1:** Statistics of effective sample sizes.

Disease Level	Number	Training Set	Prediction Set
Level 0 (healthy samples)	42	28	14
Level 1 (disease spot area < 5%)	76	51	25
Level 3 (6% < disease spot area < 10%)	65	43	22
Level 5 (11% < disease spot area < 25%)	57	38	19
Total samples	240	160	80

**Table 2:** Prediction accuracy under each model.

Principal Component/Cumulative Contribution Rate (%)	PC1	PC2	PC3
absorbance	72.345	92.368	94.522
power spectrum	69.657	89.672	93.914

**Table 3:** Prediction accuracy of each model.

Dimensions	Model	Number of Characteristic Variables	Prediction Accuracy (%)				
			Level 0	Level 1	Level 3	Level 5	Total
Near-infrared hyperspectrum	GA-BPNN	8	100	96	90.90	94.74	95
THz power spectrum	PCA-BPNN	5	100	96	95.45	94.74	96.67
THz absorbance	PCA-BPNN	6	100	92	95.45	94.74	95

**Table 4:** Prediction accuracy of each model.

Number of Characteristic Variables	Prediction Accuracy (%)				
	Level 0	Level 1	Level 2	Level 3	Total
19	99.36	95.57	96.20	97.35	97.12

## Data Availability

Data is contained within the article.
